# Transferrin Biosynthesized in the Brain Is a Novel Biomarker for Alzheimer’s Disease

**DOI:** 10.3390/metabo11090616

**Published:** 2021-09-10

**Authors:** Kyoka Hoshi, Hiromi Ito, Eriko Abe, Takashi J. Fuwa, Mayumi Kanno, Yuta Murakami, Mitsunari Abe, Takenobu Murakami, Akioh Yoshihara, Yoshikazu Ugawa, Takashi Saito, Takaomi C. Saido, Kana Matsumoto, Yoshiki Yamaguchi, Katsutoshi Furukawa, Hiroyuki Arai, Mitsuyasu Kanai, Masakazu Miyajima, Hajime Arai, Norihiro Ogawa, Hiroyasu Akatsu, Yoshio Hashizume, Hiroaki Tateno, Takashi Honda, Yasuhiro Hashimoto

**Affiliations:** 1 Department of Biochemistry, Fukushima Medical University, Fukushima 960-1295, Japan; khoshi@fmu.ac.jp (K.H.); itohrm@fmu.ac.jp (H.I.); eriko-ab@fmu.ac.jp (E.A.); tjfuwa@fmu.ac.jp (T.J.F.); 2 Department of Forensic Medicine, Fukushima Medical University, Fukushima 960-1295, Japan; kannoma@fmu.ac.jp (M.K.); ponchan@fmu.ac.jp (T.H.); 3 Department of Neurosurgery, Fukushima Medical University, Fukushima 960-1295, Japan; y-mura@fmu.ac.jp; 4 Department of Neurology, Fukushima Medical University, Fukushima 960-1295, Japan; mitzabe@gmail.com (M.A.); akioh@fmu.ac.jp (A.Y.); ugawa@fmu.ac.jp (Y.U.); 5 Division of Neurology, Department of Brain and Neurosciences, Faculty of Medicine, Tottori University, Yonago, Tottori 683-8504, Japan; maaboubou@gmail.com; 6 Laboratory of Proteolytic Neuroscience, RIKEN Center for Brain Science, Saitama 351-0198, Japan; saito-t@med.nagoya-cu.ac.jp (T.S.); takaomi.saido@riken.jp (T.C.S.); 7 Structural Glycobiology Team, RIKEN Global Research Cluster, Saitama 351-0198, Japan; k_matsu1106@yahoo.co.jp (K.M.); yyoshiki@tohoku-mpu.ac.jp (Y.Y.); 8 Institute of Development, Aging and Cancer, Tohoku University, Miyagi 980-8575, Japan; katsfuru@hotmail.com (K.F.); hiroyuki.arai.b5@tohoku.ac.jp (H.A.); 9 Division of Neurology, Mihara Memorial Hospital, Gunma 372-0006, Japan; miekanai@hotmail.co.jp; 10 Department of Neurosurgery, Juntendo University, Tokyo 113-8421, Japan; mmasaka@juntendo.ac.jp (M.M.); harai@juntendo.ac.jp (H.A.); 11 Department of Neuropathology, Fukushimura Hospital, Aichi 441-8124, Japan; norihiro@chojuken.net (N.O.); akatu@med.nagoya-cu.ac.jp (H.A.); yhashi@chojuken.net (Y.H.); 12 Cellular and Molecular Biotechnology Research Institute, National Institute of Advanced Industrial Science and Technology (AIST), Ibaraki 305-8560, Japan; h-tateno@aist.go.jp

**Keywords:** Alzheimer’s disease, cerebrospinal fluid, biomarker, transferrin, glycan-isoforms

## Abstract

Glycosylation is a cell type-specific post-translational modification that can be used for biomarker identification in various diseases. Aim of this study is to explore glycan-biomarkers on transferrin (Tf) for Alzheimer’s disease (AD) in cerebrospinal fluid (CSF). Glycan structures of CSF Tf were analyzed by ultra-performance liquid chromatography followed by mass spectrometry. We found that a unique mannosylated-glycan is carried by a Tf isoform in CSF (Man-Tf). The cerebral cortex contained Man-Tf as a major isofom, suggesting that CSF Man-Tf is, at least partly, derived from the cortex. Man-Tf levels were analyzed in CSF of patients with neurological diseases. Concentrations of Man-Tf were significantly increased in AD and mild cognitive impairment (MCI) comparing with other neurological diseases, and the levels correlated well with those of phosphorylated-tau (p-tau), a representative AD marker. Consistent with the observation, p-tau and Tf were co-expressed in hippocampal neurons of AD, leading to the notion that a combined p-tau and Man-Tf measure could be a biomarker for AD. Indeed, levels of p-tau x Man-Tf showed high diagnostic accuracy for MCI and AD; 84% sensitivities and 90% specificities for MCI and 94% sensitivities and 89% specificities for AD. Thus Man-Tf could be a new biomarker for AD.

## 1. Introduction

Alzheimer’s disease (AD) is a leading cause of dementia, accounting for two thirds of all dementia cases [1,2]. The pathological features of AD are extracellular amyloid plaques and intraneuronal neurofibrillary tangles [3]. Amyloid plaques are accumulations of abnormally folded amyloid β peptides (Aβ) [4] generated from the sequential proteolytic cleavage of amyloid precursor protein (APP) by β- and γ-secretase. APP is first cleaved by β-secretase to generate two products; a soluble APP fragment and a membrane-bound “stub” fragment called C99. C99 is further cleaved by γ-secretase at different sites to generate 40- and 42-amino-acid forms of Aβ (Aβ40 and Aβ42, respectively). Due to its hydrophobic nature and higher rate of fibrillization, Aβ42 is deposited in greater abundance in amyloid plaques than is Aβ40. Neurofibrillary tangles are another pathological feature of AD and are primarily composed of paired helical filaments consisting of hyperphosphorylated tau (p-tau) [5]. Tangle formation parallels neuronal loss and AD severity. Based on AD pathology, cerebrospinal fluid (CSF) biomarkers such as Aβ40, Aβ42, p-tau and the Aβ42/p-tau ratio [6,7] have been established. Tau/Aβ42 and p-tau/Aβ42 ratios are demonstrated to correlate well with amyloid imaging signals on positron emission tomography (PET) and would be useful for diagnosis of “preclinical” or “presymptomatic” AD [8]. Phosphorylated tau levels also increase concomitantly with tangle formation. These biomarkers established for patients with AD and mild cognitive impairment (MCI) exhibit a sensitivity and specificity reaching 85~90% [9,10]. Nevertheless, recent failures of clinical trials for potential AD therapies suggest that the disease in patients recruited for trials is already too advanced to show clinical improvement. Additional biomarkers are therefore required for diagnosis of preclinical or presymptomatic AD [11].

Tf is a single polypeptide chain with 679 amino acid residues, consisting of homologous N- and C-terminal domains [12]. Each domain has a metal binding pocket for ferric iron. Tf is *N*-glycosylated at Asn-432 and -630, with the *N*-glycans exhibiting structural diversity depending on their origin. For example, hepatic Tf is posttranslationally modified with sialylα2,6galactose-terminated biantennary *N*-glycans and then secreted into blood [13]. In contrast, a unique glycan-isoform of Tf that lacks terminal sialylα2,6galactose residues and possesses mostly *N*-acetylglucosamine (GlcNAc)-terminated *N*-glycans was identified in human CSF [14,15,16]. This isoform is secreted from CSF-producing cells in the choroid plexus [15,17] and could serve as a biomarker for altered CSF production [15,18,19]. For instance, the marker decreased in idiopathic normal pressure hydrocephalus (iNPH) [15], in which reduced CSF production was suspected. In contrast, the marker increased in patients with CSF leakage [18], in which compensatory increase of CSF production was speculated. GlcNAc-terminated Tf appears to be a functional biomarker of choroid plexus epithelium [15,17,19]. Thus, glycan structures on isoforms are “tag” for their origin and could be biomarkers of Tf producing cells.

In the present study we found another isoform of Tf carrying mannose-terminated *N-*glycans and its increase in the CSF of AD patients. The isoform was identified as a biomarker for Alzheimer’s disease (AD) and mild cognitive impairment (MCI).

## 2. Results

### 2.1. Glycan Structures of CSF Transferrin

**Figure 1:**
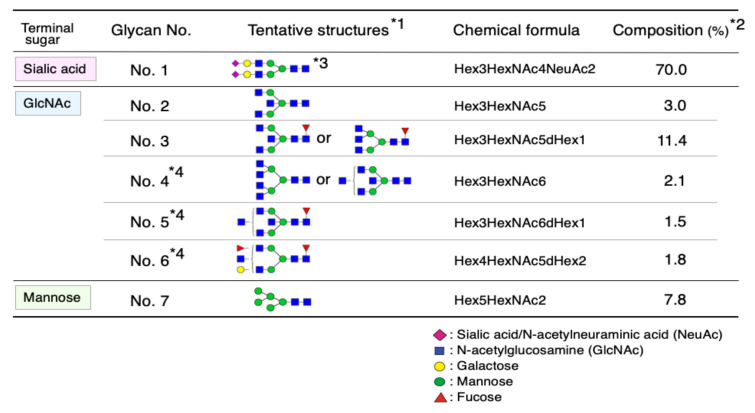
*N*-glycan structures of transferrin in human cerebrospinal fluid. Monosaccharide symbols are according to Consortium for Functional Glycomics (www.functionalglycomics.org/) (*1). Minor components (<0.6%) are not listed (*2). Glycan structure is the same as that of serum transferrin (*3). Position(s) of additional sugar(s) out of left curly brancket is not identified (*4).

We and Hoffmann et al. reported that CSF contains sialylated (Sia-Tf) and non-sialylated Tf (non-Sia-Tf) [14,15]. Due to negative charges of the sialyl residues, Sia-Tf was separated from non-sialylated Tf by anion-exchange colum chromatography [16]. Glycans of each isoform were liberated by PNGase F treatment and their composition were quantitatively analyzed by ultra-performance liquid chromatography. Glycan structures were identified by tandem mass spectrometry. As we previously reported, Sia-Tf glycan is exclusively complex-type biantennary *N-*glycan, which carries sialylgalactosyl residues at non-reducing termini (Glycan No. 1, Figure 1) [15]. In contrast to Sia-Tf, non-SiaTf carries glycans lacking the sialylgalactosyl residues; instead, the glycans carry at least one GlcNAc residue at non-reducing termini except Glycan No.7, which is a high mannose-type glycan. The GlcNAc-terminated glycans are modified with or without branching sugars; i.e., fucose attached to the innermost GlcNAc for Glycan No. 3, 5, 6 and bisecting GlcNAc for Glycan No. 2~5. These GlcNAc-terminated glycans account for 19.8% of the total *N-*glycan. A high mannose-type glycan (Glycan No. 7) constitutes 7.8% while sialo-glycan (Glycan No. 1) does 70.0% of total *N-*glycans. Tf isoforms carrying terminal mannose, GlcNAc and sialic acid are henceforth designated as Man-Tf, GlcNAc-Tf and Sia-Tf, respectively.

### 2.2. Immunoprecipitation of CSF Transferrin

**Figure 2:**
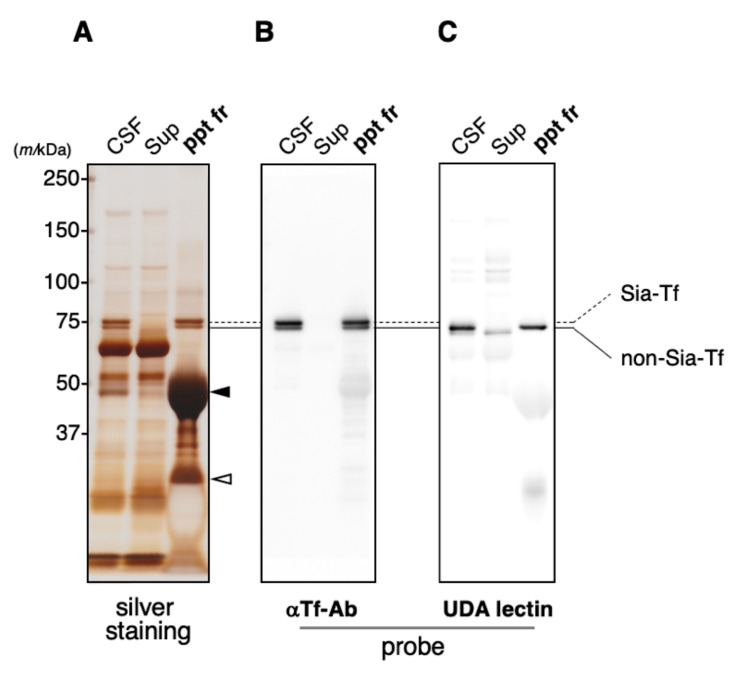
Human CSF was subjected to immunoprecipitation using anti-Tf antibody. Immunoprecipitated fraction (ppt fr) and supernatant (sup) were subjected to SDS-PAGE (**A**) followed by immuno- (**B**) and lectin-blotting (**C**). Solid and dotted lines indicate migration positions of non-Sia-Tf and Sia-Tf, respectively. Blots are probed with anti-Tf antibody (αTf-Ab) (**B**) or UDA lectin (**C**). Migration positions of immunogloblin heavy and light chains are indicated with closed and open triangles, respectively.

As glycan analysis reveals that CSF contains a unique glycan-isoform, Man-Tf, we examined whether the isoform is a sole glycoprotein carrying mannose-terminated glycans. Crude CSF was subjected to immunoprecipitation analysis to prepare precipitated fraction and supernatant. With crude CSF, doublet bands of Tf are detected around 70~75 kDa area on an immunoblot (Figure 2A,B). The upper band contains Sia-Tf and the lower one does none-Sia-Tf, as we reported previously [15,16]. The doublet is detected in precipitated fraction but not supernatant. A mannose-specific lectin, UDA, is used for detecting terminal mannose residues (Supplementary Figure S1). Lectin-blotting reveals that the lower band of doublet is a major UDA-reactive band, possibly Man-Tf. Man-Tf band is not detected in supernatant and quantitatively recovered in precipitated fraction (Figure 2C). It is also noted that a minor UDA-reactive band is detected around 70kDa area, close position to Man-Tf. The band is detected in supernatant but not precipitated fraction, suggesting that CSF contains a mannose-terminated glycoprotein(s) other than Man-Tf, but its amount is only a little. The result indicates that Man-Tf is the major mannose-terminated glycoprotein in CSF.

### 2.3. Lack of Man-Tf in CSF of Hydranencephaly Patients

**Figure 3:**
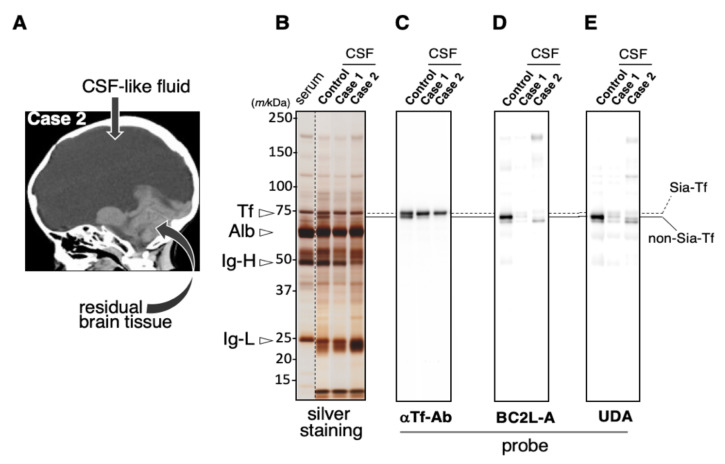
Brain CT image of a hydranencephaly patient (**A**). Control CSF and CSF-like fluid of hydranencephaly patients (Case 1 and 2) were subjected to SDS-PAGE (**B**) followed by immuno- (**C**) and lectin-blotting (**D**,**E**). Migration positions of serum Tf, albumin (Alb), immunoglobulin heavy (Ig-H) and light chains (Ig-L) are indicated with open triangles (**B**). Serum specimen is applied to a separated lane on the same SDS-PAGE gel. Solid and dotted lines indicate migration positions of non-Sia-Tf and Sia-Tf, respectively. Blots are probed with anti-Tf antibody (αTf-Ab) (**C**), BC2L-A (**D**) and UDA (**E**). Figure 3A is cited from our previous paper, J. Biochem., 2018, 164, 205–13 [17].

Hydranencephaly is a congenital malformation, in which most of cerebral hemispheres are absent and the remaining cranial cavity is filled with CSF or “CSF-like” fluid (Figure 3A) [17]. CSF of hydranencephaly and control patients were subjected to SDS-PAGE followed by immuno- and lectin-blotting. Mannose-specific lectins, UDA and BC2L-A are used for detecting mannose-terminated glycoproteins (Supplementary Figure S1). The lectin blotting reveals that Man-Tf is detected at the lower band position in control CSF while the isoform is barely detectable in CSF of hydranencephaly patients (Figure 3B–E). Minor mannose-terminated glycoproteins are also present in patient CSF. The result suggests that Man-Tf is derived from the cerebral hemispheres.

### 2.4. Expression of Tf mRNA and Tf protein in the Cerebral Cortex

**Figure 4:**
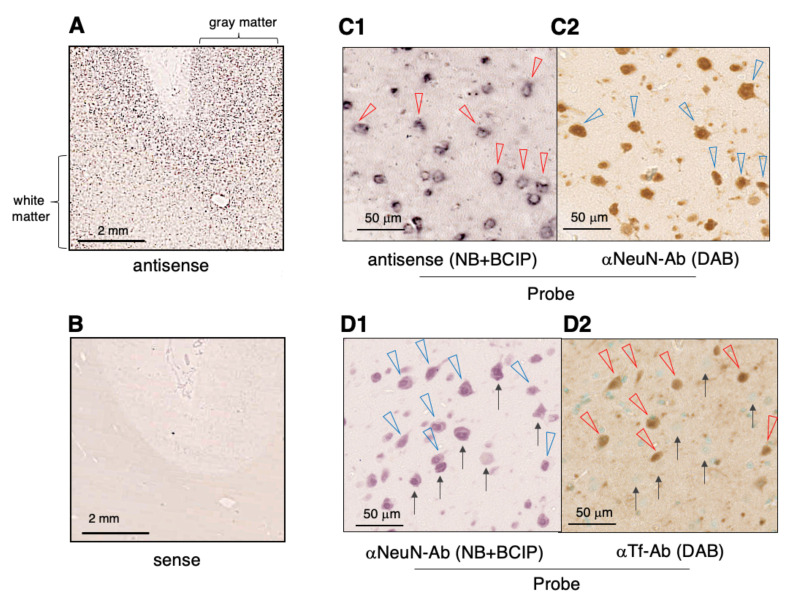
*Tf mRNA* expression was examined by *in situ* hybridization. The lateral lobe sections were hybridized with anti-sense (**A**) and sense (**B**) probes, and then visualized with a mixture of 4-nitroblue tetrazolium chloride and 5-bromo-4-chloro-3-indoyl-phosphate mixture (NBT/BCIP). In a high-power field, *Tf mRNA*-positive cells are indicated with red triangles (**C1**). The corresponding area on the mirror image section was stained with anti-NeuN antibody (αNeuN-Ab), and the antigens were visualized with the Immpact DAB Substrate (DAB). NeuN-positive neuron-like cells are indicated with blue triangles (**C2**). Tf protein expression was examined by immunohistochemistry using anti-Tf antibody (αTf-Ab). In a high-power field of the lateral cortex, many neuron-like cells are stained by anti-NeuN antibody, visualizing with NBT/BCIP (**D1**, blue triangles and arrows). The corresponding area on the mirror image section is stained by anti-Tf antibody, visualizing with DAB. Tf-positive cells are indicated with red triangles, while NeuN-positive, but Tf-negative, cells are indicated with arrows (**D2**, right panel). The cellular nuclei are stained with methyl green.

As CSF analysis of hydranencephaly patients suggests that Man-Tf is derived from the cerebrum, we analyzed the expression of *Tf mRNA* and Tf protein in the temporal lobe. *In situ* hybridization reveals positive signals in the grey matter and the signals are more intense than white matter (Figure 4A,B). In a high magnification figure of the grey matter, significant signals are detected in large neuron-like cells (Figure 4C1; red triangles). These cells are stained with anti-NeuN (a neuron marker) antibody on a corresponding mirror image section (Figure 4C2; blue triangles), suggesting that these neurons express *Tf* mRNA. In the white matter, the signals are detected with some small cells, possibly oligodendrocytes (data not shown). Expression of Tf protein in the cerebral cortex was also examined by immunohistochemistry using anti-Tf and anti-NeuN antibodies. Multiple neurons are stained with anti-NeuN antibody (Figure 4D1; blue triangles and arrows), while some neurons show evidence of Tf protein expression (Figure 4D2; red triangles) but others did not (arrows). This result suggests that Tf protein expression varies among cortical neurons even though most of them express *Tf mRNA*. It was also noted that some oligodendrocyte-like cells express Tf protein in the white matter (data not shown).

### 2.5. Glycan Analysis of Tf in the Cerebral Cortex

**Figure 5:**
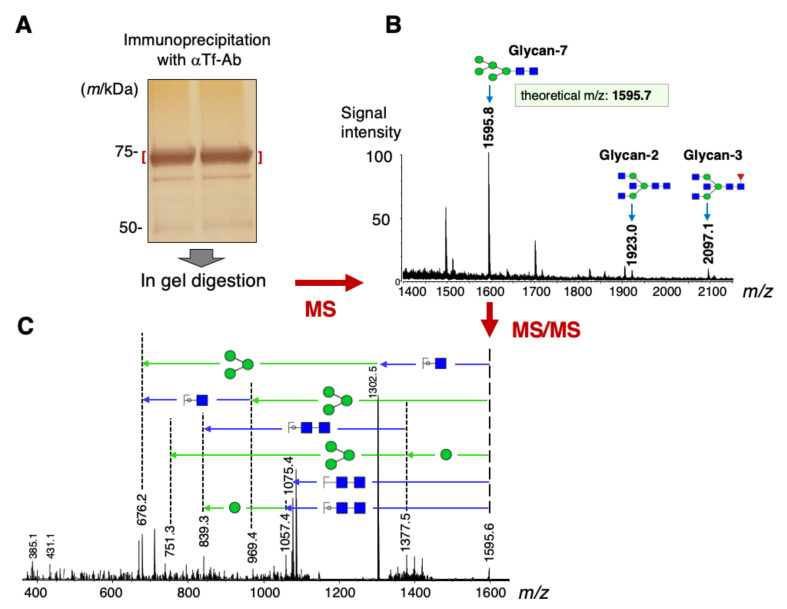
Tf was purified from detergent extracts of human occipital cortex by immunoaffinity column chromatography and subjected to SDS-PAGE (**A**). Glycans were liberated from Tf by in-gel digestion with PNGase F. Liberated glycans are reduced, permethylated and then subjected to matrix-assisted laser desorption/ionization time-of-flight mass spectrometry (MS) (**B**). Secondary fragment ions derived from the ion at m/z 1595.8 were analyzed on tandem mass spectrometry (MS/MS) (**C**).

As Tf isoforms are cell-type specific, glycans of cortex Tf is analyzed by mass spectrometry. Tf was purified from detergent extracts of human occipital cortex by immunoaffinity column chromatography. Purified Tf showed an intense band on SDS-PAGE (Figure 5A), which were subjected to in-gel digestion with PNGase F to liberate *N*-glycans. The glycans were reduced, permethylated and then subjected to MALDI/TOF/MS (MS). The most intense signal is detected at *m/z* 1595.8 (Figure 5B), close to the theoretical *m/z* of Glycan No. 7, being 1595.7. Tandem mass spectrometry (MS/MS) indicates that the fragmentation pattern derived from the 1595.8 ion is consistent with that of Glycan No.7 (Figure 5C). In addition to the Glycan-7 signal, possible ions of GlcNAc-terminated *N-*glycans (Glycan No. 2 and 3) are detected on MALDI/TOF/MS at *m/z* 1923.0 and 2097.1, respectively (Figure 5B). Signals corresponding to other GlcNAc-terminated glycans (Glycan No. 4–6) are not detectable. These results suggest that Man-Tf is the most abundant isoform (ca. 90%) in the occipital cortex.

### 2.6. Man-Tf Levels in the CSF of Patients with Different Neurological Diseases

**Figure 6:**
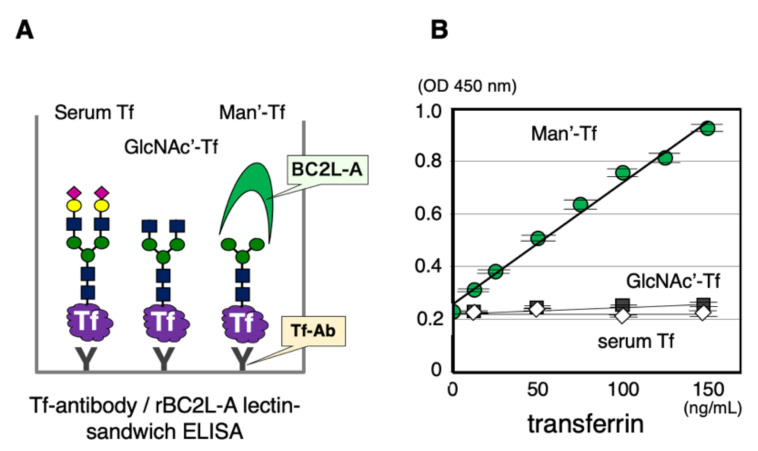
Tf glycan isoforms were quantified by anti-Tf antibody/rBC2L-A lectin-sandwich ELISA. The isoforms are captured on a microtiter plate by using anti-Tf antibody (Tf-Ab) (**A**). The mannose-terminated isoform (Man’-Tf) is quantified with rBC2L-A lectin (**B**). No significant signal is detected with Sia-Tf and GlcNAc’-Tf.

Man-Tf is the major isoform in the occipital cortex and possibly secreted by neurons. On the premise that Man-Tf could be a biomarker for neurodegeneration, its levels were analyzed in the CSF of patients with neurological diseases. For quantification, we newly developed an antibody/lectin-sandwich ELISA (Figure 6A). Anti-Tf antibody is coated on a microtiter plate for capturing all isoforms and then mannose-terminated Tf can be specifically detected with rBC2L-A lectin. The glycan-specificity of ELISA is examined with serum Tf and authentic isoforms carrying truncated glycans, GlcNAc’-Tf and Man’-Tf (Supplementary Figure S1). The calibration curve of Man’-Tf is linear in the range of 10~150 ng/mL while serum Tf and GlcNAc’-Tf show only a background level signal (Figure 6B). With Man’-Tf, intra- and inter-assay reproducibilities were 7% and 11%, respectively, with a spike recovery of 91%. Dilution linearity of CSF was observed in the 0.5~5 μL range.

**Figure 7:**
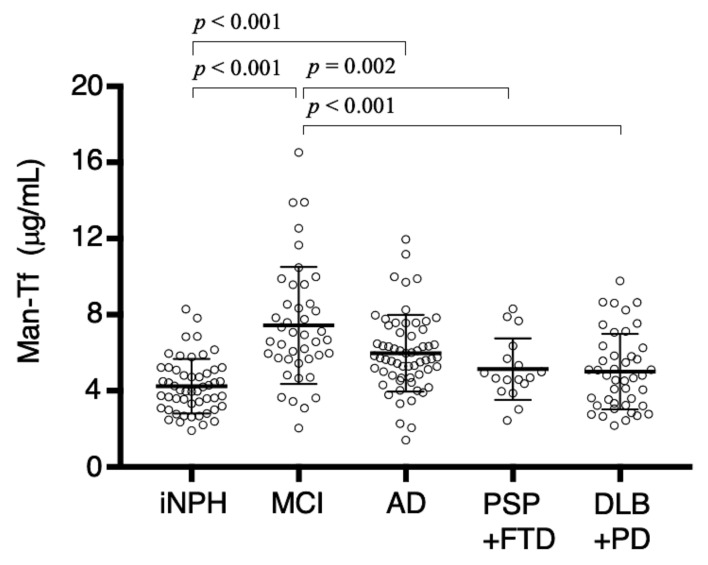
Man-Tf levels are quantified in CSF of neurological disease: iNPH, idiopathic normal pressure hydrocephalus; MCI, mild cognitive impairment; AD, Alzheimer’s disease; PSP, progressive supranuclear palsy; FTD, frontotemporal degeneration; PD, Parkinson’s disease; DLB, dementia with Lewy bodies. Man-Tf data show normal distribution in iNPH, AD, PSP+FTD groups, but not in others. Multiple comparisons were assessed by Kruskal-Wallis method followed by Bonferroni correction. Significant differences are indicated with p values.

By the use of ELISA, we quantified Man-Tf levels in the CSF of patients with neurological diseases such as AD, MCI, other tauopathies and synucleinopathies. CSF of iNPH patients was also analyzed as a control, because it manifests dementia due to hydrocephalus but does not include neurodegeneration. Kruskal-Wallis multiple comparison test reveals that Man-Tf levels are significantly higher in MCI (7.44 ± 3.07 μg/mL, *p* < 0.001) and AD (5.97 ± 2.01 μg/mL, *p* < 0.001) than in iNPH (4.24 ± 1.43 μg/mL) (Figure 7). MCI shows higher levels than AD, but difference is not significant. The result suggests that Man-Tf increment is an early event in AD pathology. A slight, but not significant, increase is detected in CSF samples from patients with tauopathies, PSP and FTD, and synucleinopathies, PD and DLB.

**Figure 8:**
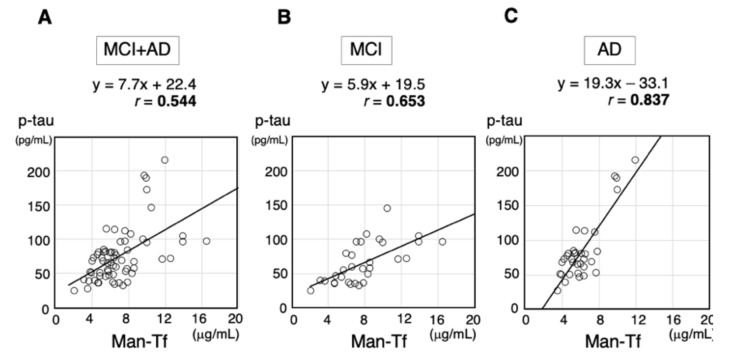
Correlation coefficients for p-tau and Man-Tf; MCI+AD (**A**), MCI (**B**) and AD (**C**).

As these findings suggest that elevated Man-Tf levels are associated with AD pathology, we examined the correlation between Man-Tf and AD core markers such as p-tau, tau, Aβ40 and Aβ42 in CSF. A scatter diagram for Man-Tf and p-tau reveals a correlation between the two parameters in MCI+AD group (*r* = 0.544) (Figure 8A). MCI and AD groups show a moderate (*r* = 0.653) and strong (*r* = 0.837) correlation, respectively (Figure 8B,C). The slope of the regression line for AD (19.3) is steeper than that of MCI (5.9), suggesting that the slope increases in line with disease progression. In addition to p-tau, we analyzed other AD core markers such as tau, Aβ40 and Aβ42. Man-Tf correlated weakly with these markers (0.144~0.284) except for Aβ40 in MCI (*r* = 0.457) and tau in AD (*r* = 0.535), where the correlation was moderate. As we reported previously, CSF Sia-Tf is possibly derived from blood. Its levels were not different between AD (18.37 ± 7.14 μg/mL) and iNPH (18.60 ± 7.13 μg/mL).

### 2.7. Expression of p-tau and Man-Tf in Hippocampus of AD Patients

**Figure 9:**
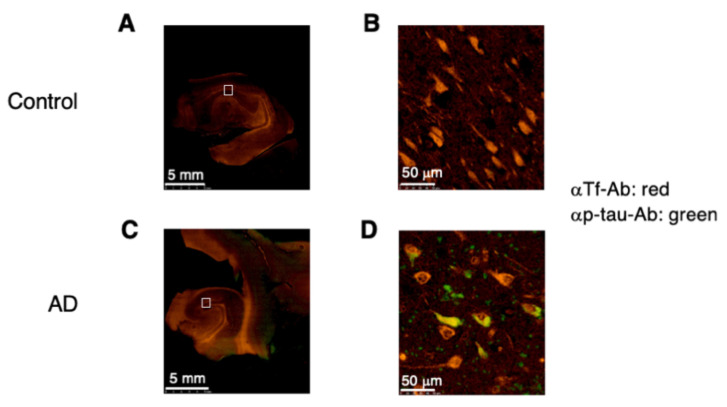
Human hippocampal sections from control (normal; **A**,**B**) and AD (**C**,**D**) brains was co-stained with anti-Tf antibody (αTf-Ab; red) and anti-p-tau antibody (αp-tau-Ab; green). Images were scanned by NanoZoomer and analyzed by NDP.view2 Plus Image viewing software U12388-01.

P-tau is secreted from affected neurons and its CSF levels are increased along with AD progression. As Man-Tf concomitantly increase with p-tau in CSF, we examine colocalization of Tf (red) and p-tau (green) in hippocampal neurons by fluorescent immunohistochemistry. Hippocampal grey matter is stained with anti-Tf antibody in control and AD sections (Figure 9A,C). In a high-power field, Tf-positive staining is evident in many neuron-like cells in both cases (Figure 9B,D). In AD brains, some neuron-like cells co-stain with anti-p-tau antibody, AT8 (Figure 9D). Small areas of neuropil stain positively for p-tau alone. These results suggest that Tf is actively produced by hippocampal neurons in control and AD brains, and that some neurons co-express p-tau in AD pathology.

### 2.8. Diagnostic Accuracy of Man-Tf and Its Combination with P-tau

**Figure 10:**
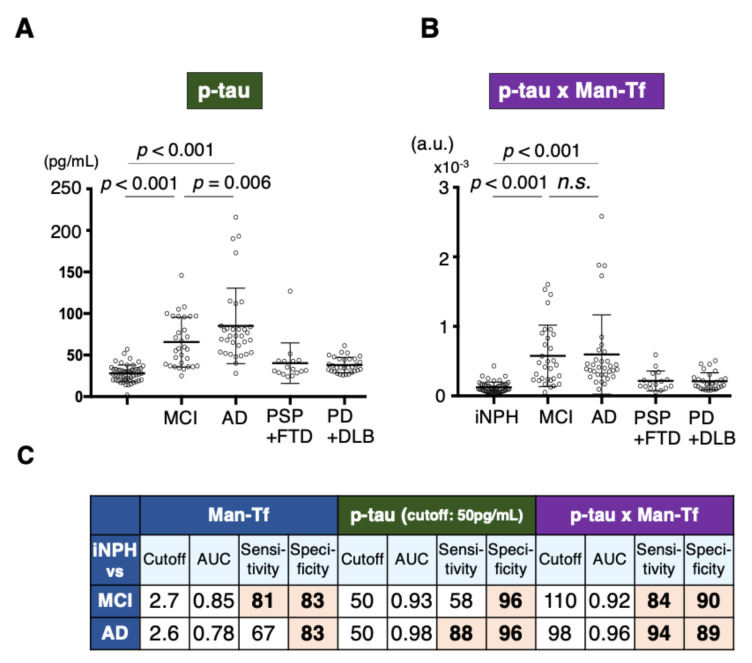
Levels of p-tau (**A**) and p-tau x Man-Tf (**B**) are indicated. P-tau data show normal distribution in iNPH group, but not in others. Data of p-tau x Man-Tf do not show normal distribution in all groups. Multiple comparisons are assessed by Kruskal-Wallis method followed by Bonferroni correction. Sensitivities, specificities, AUCs, and cutoff values of markers are indicated (**C**).

We next examined the diagnostic accuracy underlying the use of Man-Tf to differentiate MCI and AD from iNPH. Man-Tf differentiated MCI with 81% sensitivity and 83% specificity, and AD with 67% sensitivity and 83% specificity, thus showing low diagnostic accuracy for AD (Figure 7 and Figure 10C). The diagnostic accuracy of p-tau was also examined with a cutoff value of 50 pg/mL, which is the manufacturer’s recommendation. P-tau differentiated AD from iNPH with high sensitivity and specificity, but MCI with low sensitivity (59%) (Figure 10A,C). Combined Man-Tf and p-tau (p-tau x Man-Tf) use was examined for its diagnostic accuracy because of their concomitant increase in the disease progression. The product exhibited high diagnostic accuracy for MCI and AD (Figure 10B,C). Thus, p-tau × Man-Tf could be a new biomarker for differentiating MCI and AD from iNPH.

## 3. Discussion

We demonstrated here that CSF contains Tf glycan-isoforms; Sia-Tf, GlcNAc-Tf and Man-Tf. Hydranencephaly patients lack CSF Man-Tf, indicating that the glycoform is derived from the cerebrum. In the lateral cerebral cortex, NeuN-positive neurons express *Tf* mRNA and Tf protein. MS analysis revealed that Man-Tf is the major isoform in the occipital cortex. These results suggest that CSF Man-Tf is, at least partly, derived from the cerebral cortex, and possibly from neurons. We therefore examined whether this isoform could be a biomarker of neurodegeneration. Man-Tf levels were analyzed in the CSF of patients with neurological diseases such as AD, MCI and other tauopathies and synucleinopathies as well as iNPH. Multiple comparison analyses revealed that Man-Tf levels are significantly higher in MCI than in other neurodegenerative disorders and iNPH (Figure 7). It has been reported that clinically suspected iNPH is often comorbid with AD pathology [20,21], but the iNPH patients we analyzed were shunt responsive and classified as “definite” iNPH. In addition, CSF p-tau levels in 50 out of 52 iNPH patients were less than the cutoff value, 50 pg/mL, while the other two cases were 52 and 57 pg/mL (Figure 10A). The low levels of CSF p-tau and shunt responsiveness suggest that the iNPH patients we analyzed have minimal AD pathology, if any. Thus, iNPH was used as a control in our comparative analyses.

Castillo et al. reported that *Tf* mRNA levels of AD brain increased 1.93- and 1.15-fold in the temporal and frontal cortices, respectively, comparing with control brain [22]. This suggests that *Tf* upregulation correlates with AD pathology. On this basis we analyzed the correlation between Man-Tf and AD core markers in CSF. Among them, p-tau correlated well with Man-Tf in MCI (*r* = 0.653) and AD (*r* = 0.837) (Figure 8B,C). In addition, AD patients showed the steeper slope of the regression line than MCI patients, suggesting that the slope is associated with the disease progression. High correlation of CSF p-tau and Man-Tf appears consistent with the observation that some of hippocampal neurons were co-stained with antibodies against the markers (Figure 9D). Both Man-Tf and p-tau were elevated in MCI and AD, leading to the notion that the product of Man-Tf and p-tau (p-tau × Man-Tf) could serve as a marker for MCI and AD. Indeed, the product differentiates MCI and AD from iNPH with high accuracy (Figure 10C,D), suggesting that Man-Tf is a new biomarker related to AD pathology.

Alzheimer’s disease pathology is characterized by amyloid plaques and neurofibrillary tangles, which are composed of abnormal aggregates of Aβ and p-tau, respectively. The accumulation of aggregates triggers unfolded protein response in the endoplasmic reticulum (ER). In this response, some of misfolded proteins are re-folded correctly to move to Golgi apparatus for further processing, otherwise misfolded proteins are ubiquitinated and translocated into cytosol for proteasome-dependent degradation (ER stress response). In the system, high mannose-type glycan on misfolded protein is recognized by endogenous mannose-binding lectins or lectin-like proteins. Accordingly, proteins including Man-Tf carrying high mannose-type glycans may increase in ER stress response. In the present study we demonstrate that CSF Man-Tf is elevated in MCI and AD. This may be due to ER stress of neurons. In this context, it is interesting to analyze Man-Tf levels in other ER stress-inducing neuronal diseases.

CSF biomarkers such as Aβ42 and p-tau facilitate the accurate diagnosis of AD, but the invasiveness of lumbar punctures limits the number of patients tested [11]. Current trials aimed at quantifying plasma p-tau offer promise as laboratory tests [23,24]. While Moscoso et al. reported that plasma p-tau181 is a marker for predicting and monitoring neurodegeneration and cognitive decline in AD [25]. Tatebe et al. reported that the plasma p-tau concentration is only 0.1% of that of the CSF [23], thus necessitating sensitive measurement methods such as multiplex immunoassay [26]. A potential advantage of Man-Tf may be its high concentration in CSF (2~10 μg/mL). Given that Man-Tf is translocated from CSF into plasma similar to p-tau, it would be detectable with the antibody/lectin-ELISA we have developed. Quantifying plasma or serum Man-Tf could be incorporated into routine clinical practice and make it possible to carry out repeat sampling throughout the course of AD progression.

Taniguchi et al. reported that a glycan-isoform of Tf carrying wheat germ agglutinin (WGA) epitopes is decreased in the CSF of AD patients and could serve as a biomarker for the disease [27]. Based on the results of isoelectric focusing experiments, they speculated that the decreased signal is due to loss of WGA epitopes on Tf isoform, i.e., loss of sialyl residues on Sia-Tf. However, we did not detect a decrease of Sia-Tf in the CSF of AD patients. Inconsistencies in the results could be due to the complexity of Tf glycan structures and their different reactivities to lectins used (https://acgg.asia/lfdb2/). Glycan changes to Tf in the context of AD need to be chemically confirmed in future studies.

## 4. Limitations

The study is limited to Japanese patients. Even though the study is carried out with multicenter platform, the number of patients recruited is limited.

## 5. Materials and Methods

### 5.1. Patients

Patients with AD and MCI were consecutively recruited from Fukushima Medical University; AD, MCI, PSP, FTD, DLB, PD from Tohoku University; iNPH from Juntendo University and Mihara Menorial Hospital. For each disease, the number of patients, age (mean ± S.D.), male/female distribution, MMSE and biomarker levels of Aβ40, Aβ42, p-tau and tau are listed in Table 1. Disease diagnosis was based on the following criteria: AD, “The diagnosis of dementia due to Alzheimer’s disease: Recommendations from the National Institute on Aging-Alzheimer’s Association workgroups on diagnostic guidelines for Alzheimer’s disease.“ [2]; MCI, “The diagnosis of mild cognitive impairment due to Alzheimer’s disease: recommendations from the National Institute on Aging-Alzheimer’s Association workgroups on diagnostic guidelines for Alzheimer’s disease” [28]; progressive supranuclear palsy (PSP), “Accuracy of clinical criteria for the diagnosis of progressive supranuclear palsy” [29]; frontotemporal degeneration (FTD), “Frontotemporal lobar degeneration: a consensus on clinical diagnostic criteria” [30]; dementia with Lewy bodies (DLB) “Diagnosis and management of dementia with Lewy bodies: Fourth consensus report of the DLB Consortium.” [31]; Parkinson’s disease (PD), “the Queen Square Brain Bank criteria for the diagnosis of Parkinson’s disease” (https://www.mims.ie/news/queen-square-brain-bank-qsbb-criteria-for-pd-diagnosis-02-04-2013/); idiopathic normal pressure hydrocephalus (iNPH), “Guidelines for management of idiopathic normal pressure hydrocephalus, second edition” [32]. Recruited iNPH patients did not show increase of AD core-biomarkers in CSF and were classified as “definite” iNPH due to improved symptoms after shunt surgery. 

### 5.2. Glycan Analysis of Transferrin Purified from CSF and Cerebral Cortex

CSF for Tf glycan analysis was pooled from patients with unruptured aneurysm, trigeminal neuralgia and facial spasm, but without apparent neurodegenerative diseases. CSF was filtered, dialyzed and then purified by column chromatographies, using Blue Sepharose, anion-exchange, protein G and gel filtration columns [16]. *N*-glycans attached to the Tf isoforms were liberated with PNGase F (#4450, Takara Bio Inc., Shiga, Japan) and then labeled with a Glycowarks *Rapi*Fluor-MS *N*-glycan kit (Waters Corporation, Milford, MA, USA) according to the manufacturer’s instructions. Labeled *N*-glycans were analyzed by ultra-performance liquid chromatography with fluorescence detection followed by MS/MS or MS^E^ technology according to the manufacturer’s protocol (Waters Corporation). The relative quantity of each *N*-glycan was estimated based on the fluorescence intensity of the corresponding peak. Tissue Tf was purified from human cerebral cortex by immunoaffinity column chromatography. Briefly, Tf was extracted from frozen occipital cortex (ca. 1g) with 1% NP-40 in Tris-HCl buffer (pH 7.4). Extracts were purified on a Tf antibody-immobilized column. The purified Tf was applied to SDS-PAGE and then stained with a silver stain MS kit (299-58901, FUJIFILM Wako Pure Chemical Corporation, Osaka, Japan). Tf bands were subjected to in-gel digestion with PNGase F (#4450, Takara Bio Inc., Shiga, Japan) to release *N*-glycans, which were reduced, permethylated and subjected to mass spectrometry.

### 5.3. SDS-PAGE and Blotting Analyses

CSF samples were dissolved in Laemmli buffer without 2-mercaptoethanol, boiled for 3 min and loaded on SDS-polyacrylamide gels (194–1502, FUJIFILM Wako) [33]. After SDS-PAGE, protein bands were visualized with a Silver Stain II kit (FUJIFILM Wako). For immunoblotting, proteins separated on gels were transferred to nitrocellulose membranes. The membranes were blocked in 3% skim milk, incubated sequentially with anti-transferrin antibody (Bethyl Laboratories, Montgomery, TX, USA) and horseradish peroxidase-labeled anti-goat IgG (Jackson ImmunoResearch Laboratories, West Grove, PA, USA), and developed using a SuperSignal West Dura Chemiluminescence Substrate Kit (Pierce Biotechnology, Rockford, IL, USA). For lectin blotting, membranes were blocked in 1% BSA and incubated with a mannose-binding lectin, rBC2L-A (026-18691, FUJIFILM Wako). Prior to use, rBC2L-A lectin was biotinylated using Ez-Link NHS-Biotin (#20217, Thermo Fisher Scientific, Waltham, MA, USA). Membranes were further incubated with horseradish peroxidase-labeled streptavidin (SA00001-0, Cosmo Bio Co., Tokyo, Japan), and developed. Biotinylated *Urtica dioica* Lectin (UDA) (BA-8005-1, Cosmo Bio Co.) and biotinylated Concanavalin A (ConA) (BA-1104-5, Cosmo Bio Co.) were also used for mannose-binding lectins. 

### 5.4. Preparation of Authentic Tf Isoforms

Serum Tf was digested sequentially with glycosidases; generating galactose-terminated Tf (Gal’-Tf), GlcNAc-terminated Tf (GlcNAc’-Tf), and mannose-terminated Tf (Man’-Tf). Their reactivities to rBC2L-A, UDA and ConA were examined by lectin-blotting (Supplementary Figure S1). The isoforms were used as standards for ELISA.

### 5.5. Immunoprecipitation of CSF Tf

Anti-Tf antibody was immobilized on Protein A/G agarose beads (abcam, Cambridge, UK) according to manufactural protocol. The beads were incubated with CSF for 4 h at 4 °C and the supernatant was removed. The beads were washed three times with phosphate buffered saline. Antigen-antibody complex was eluted by heating the beads at 95 °C in a SDS-PAGE loading buffer. The supernatant was removed as a precipitated fraction.

### 5.6. ELISA for CSF Proteins

CSF samples was aliquoted and stored at −80 °C until use. Repeated freeze-thawing was avoided, less than two times. Each assay was performed in triplicate. For quantifying Man-Tf, a lectin-ELISA was developed according to Shirotani et al. [33] with slight modification. Briefly, a 96-well plate (C8 Maxisorp Nunc-Immuno Module plate, Nunc, Roskilde, Denmark) was coated with a rabbit anti-Tf antibody (1μg/mL) (A0061, Dako Ltd./Agilent Technologies, Inc., Santa Clara, CA, USA) in 100 mM carbonate buffer at 4 °C overnight. The plate was washed with Tris buffered saline (TBS) and then blocked at room temperature for 1 h with 10% N101 (S410-0301, NOF Corp., Tokyo, Japan) in TBS. The plate was washed with TBS containing 0.05% Tween 20 (#1706531, Bio-Rad Laboratories, Inc., Hercules, CA, USA) (TBST). The CSF samples were pre-treated at 55 °C for 1 h in the presence of 10~20 μL of phosphate buffered saline (PBS) containing 0.6% 2-mercaptoethanol (#1610710, Bio-Rad Laboratories, Inc.) and 0.003% SDS (S0588, Tokyo Chemical Industry Co., Ltd., Tokyo, Japan). The sample solution was appropriately diluted with TBST, applied to the plate and incubated at 4 °C overnight. The plate was washed three times with TBST. The target molecule, Man-Tf, captured on the plate was incubated with biotinylated rBC2L-A lectin (1μg/mL) in TBST containing 10 mM CaCl_2_ (TBST-CaCl_2_) at room temperature for 2 h. The plate was washed two times with TBST-CaCl_2_. Horseradish peroxidase-labeled streptavidin (50 ng/mL) (N100, Thermo Fisher Scientific, Waltham, MA, USA) in TBST-CaCl_2_ was added to the plate and incubated for 2 h at room temperature. The plate was washed two times with TBST-CaCl_2_. TMB Micro well Peroxidase Substrate System (#50-76-11, Kirkegaard and Perry Laboratories, Inc., Gaithersburg, MD, USA) was added to the plate. Color development was stopped by adding 1N phosphoric acid. Absorbances were measured at 450 nm by a Varioskan LUX multimode microplate reader (Thermo Fisher Scientific, Waltham, MA, USA). Assays were performed in triplicate. For quantifying Sia-Tf and GlcNAc-Tf, the lectin probes used were SSA (197-10371, FUJIFILM Wako) and PVL (Medical and Biological Laboratories Co., Ltd., Nagoya, Japan), respectively [33]. Total Tf was quantified with a Human Transferrin ELISA Quantitation Set (E80-128-23, Bethyl Laboratories). AD core markers were assayed by LSI Medience Corporation (Tokyo, Japan), using the following ELISA kits: Phinoscholar hTAU (10-992, Nipro Parma Corporation, Osaka, Japan) for (total) tau; Phinoscholar pTAU (10-994, Nipro) for p-tau (181); Human βAmyloid (1-40) ELISA Kit Wako II (298-64601, FUJIFILM Wako) for Aβ40; Human βAmyloid (42) ELISA Kit Wako High Sensitive (292-64501, FUJIFILM Wako) for Aβ42.

### 5.7. Preparation of Tissue Sections and Pairs of Mirror Image Sections

Brain samples were fixed in 4% paraformaldehyde/PBS for 24 h, and then paraffin-embedded, sectioned at 3 or 5 μm, and mounted on glass slides. Pairs of “mirror image” sections were prepared as follows [34,35]. Two serial sections (5-μm thick) were prepared from brain tissue. One section was inverted, floated on PBS and then mounted on a glass slide (inverted section). The second section was mounted on another glass slide (non-inverted section) and the pair of mirror-image sections then subjected to histological analysis. The inverted section was stained with anti-NeuN antibody and the non-inverted section was subjected to either anti-Tf antibody staining or in situ hybridization.

### 5.8. In Situ Hybridization (ISH)

Digoxigenin (DIG)-labeled cRNA probes were prepared based on the human transferrin ORF sequence (NM_001063.3). Target sequences were amplified by PCR using cloned Tf cDNA (Origene, Rockville, MD). The forward and reverse primers used were 5’-gggtaatacgactcactatagggctccacccttaaccaatacttc-3’ and 5’-gtgattaaccctcactaaagggaatcccttctcaaccagacacc-3’, respectively, with T7 and T3 promoter sequences underlined. Hybridization probes were prepared by in vitro transcription using a DIG-labeling kit (11175033910, Roche Diagnostics K.K., Tokyo, Japan). Deparaffinized sections were pretreated with proteinase K (FUJIFILM Wako) and acetic anhydride. DIG-labeled cRNA probes were diluted to 500 ng/mL in hybridization buffer (composed of 50% formamide, 30 mM trisodium citrate buffer (pH7.0) containing 300 mM NaCl, Denhardt’s solution (10727-74, Nacarai Tesque, Kyoto, Japan), 100 µg/mL salmon sperm DNA (F012, Funakoshi Co., Ltd., Tokyo, Japan) and 300 µg/mL yeast tRNA (11585762001, Roche Diagnostics K.K.) and denatured at 99 °C for 5 min. Proteinase K-treated sections were pre-incubated with hybridization buffer at 60 °C for 30 min and then incubated with cRNA probe in hybridization buffer at 60 °C overnight. After extensive washing, sections were incubated with alkaline phosphatase-conjugated anti-DIG antibody (A1054, Roche Diagnostics) in an incubation buffer (C7594, Roche Diagnostics) for 1 h at room temperature. After washing, sections were incubated in 100 mM Tris-HCl buffer (pH9.5) containing 100 mM NaCl for 10 min. Probe RNA was visualized with a 4-nitroblue tetrazolium chloride (NBT)/5-bromo-4-chloro-3-indoyl-phosphate (BCIP) mixture (11681451001, Roche Diagnostics K.K.) in the dark at room temperature. To stop color development, sections were washed with a 10 mM Tris-HCl buffer (pH8.0) containing 1 mM EDTA. Mirror-image sections were stained with anti-NeuN antibody as described below.

### 5.9. Immunohistochemistry

Sections for immunohistochemistry were autoclaved in sodium citrate buffer (pH6.0) for 15 min at 121 °C for antigen retrieval. Endogenous peroxidase was quenched with BLOXALL (SP-6000-100, Vector Laboratories, Burlingame, CA, USA) for 10 min at room temperature. For detecting Tf, sections were incubated overnight at 4 °C with 0.1 µg/mL of goat polyclonal antibody to human Tf (A80-128P, Bethyl Laboratories). After extensive washing, sections were incubated with 1.0 µg/mL of horse radish peroxidase-labeled (HRP) anti-goat IgG (AB_2340390, Jackson Immunoresearch Laboratories, Inc., West Grove, PA, USA) at room temperature for 1.5 h. Tf antigen was visualized with the Immpact DAB Substrate (SK-4105, Vector Laboratories). To stop signal development, sections were washed with distilled water. Methyl green was used for counterstaining nuclei. Mirror-image sections were stained with anti-NeuN antibody (ab177487, Abcam, Cambridge, UK) in PBS containing 3% BSA at 4 °C overnight. After extensive washing, the sections were incubated with alkaline phosphatase-conjugated goat anti-rabbit antibody (ab6722, Abcam) in PBS containing 3% BSA for 1.5 h at room temperature. NeuN antigen was visualized with NBT/BCIP mixture in the dark at room temperature. On mirror-image sections for in situ hybridization, NeuN antigen was visualized with the Immpact DAB Substrate using HRP-labeled goat anti-rabbit IgG as secondary antibody (ab6721, Abcam). For immunofluorescence microscopy, paraffin-embedded hippocampal sections from normal and AD brains (purchased from FUJIFILM Wako, Japan) were subjected to antigen retrieval by autoclaving (121 °C for 5 min) followed by tyramide signal amplification (PerkinElmer Life Sciences, Waltham, MA, USA) as described previously [36]. The brain sections were immunostained using antibodies specific to phosphorylated tau (AT8; #90206, Innogenetics, Ghent, Belgium) and to anti-Tf antibody (A0061; DAKO). Alexa Fluor 594-labeled anti-mouse IgG and Alexa Fluor 488-labeled anti-rabbit IgG were used as secondary antibodies, respectively. All images were scanned by NanoZoomer and analyzed by NDP.view2 Plus Image viewing software U12388-01 (Hamamatsu Photonics, Hamamatsu, Japan).

### 5.10. Statistical Analyses

Statistical analyses were performed using SPSS (version 26). Normality of each data was examined by the Shapiro–Wilk test. Significant differences among multiple comparisons were assessed by Kruskal-Wallis method followed by Bonferroni correction. Receiver operating characteristic (ROC) analyses were used for evaluating the predictive accuracy of the model. Cutoffs were determined by Youden index and areas under the curve (AUCs) were used for estimating predictive accuracy.

## Figures and Tables

**Table 1:** Characteristics of Patients.

Disease	Age * (Years)	Patient Number	Gender (M/F)	MMSE *	Aβ40 * (pg/mL)	Aβ42 * (pg/mL)	p-tau * (pg/mL)	Tau * (pg/mL)
**iNPH:** idiopathic normal pressure hydrocephalus	77.5 ± 6.9	52	30/22	22.4±5.5	3600 ± 1260	312 ± 207	27.9 ± 10	191 ± 48
**MCI:** mild cognitive impairment	76.0 ± 6.9	42	19/23	26.9 ± 1.7	7260 ± 3180	630 ± 268	65.7 ± 30	428 ± 220
**AD:** Alzheimer’s disease	73.5 ± 8.7	61	27/34	20.4 ± 4.2	5620 ± 2750	537 ± 182	85.1 ± 46	652 ± 260
**PSP:** progressive supranuclear palsy	69.6 ± 5.7	7	4/3	n.d.	5810 ± 2890	611 ± 337	33.9 ± 6.8	194 ± 64
**FTD:** frontotemporal degeneration	60.7 ± 4.1	10	5/5	n.d.	n.d.	n.d.	45.5 ± 32	360 ± 290
**DLB:** dementia with Lewy bodies	66.8 ± 2.6	9	6/3	n.d.	n.d.	n.d.	39.3 ± 4.4	184 ± 75
**PD:** Parkinson’s disease	68.2 ± 8.5	34	13/21	n.d.	n.d.	n.d.	37.8 ± 9.8	187 ± 75

* mean ± S.D.; n.d., not determined.

## Data Availability

All data is contained within the article or Supplementary Materials.

## Supplementary Materials

The following is available online at https://www.mdpi.com/article/10.3390/metabo11090616/s1, Figure S1: Binding specificities of mannose-binding lectins.
