# Supplementary figures and images for: Transferrin Biosynthesized in the Brain Is a Novel Biomarker for Alzheimer’s Disease
Source: Metabolites. 2021 Sep 10;11(9):616. doi: 10.3390/metabo11090616 (PMC8470343; doi:10.3390/metabo11090616)

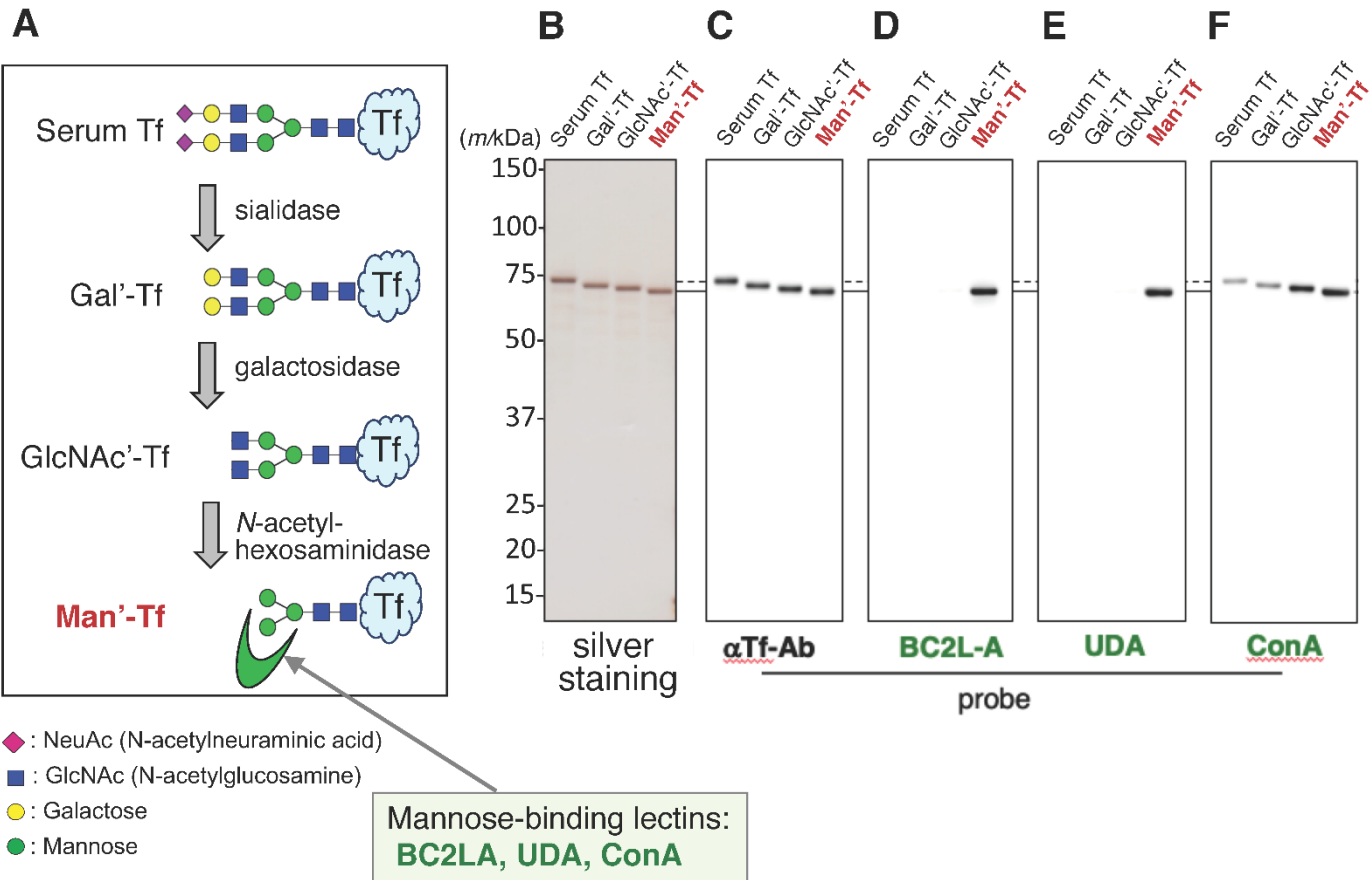

**Figure S1.** Binding specificities of mannose-binding lectins.

Supplement: Supplementary file 1 [file metabolites-11-00616-s001.zip › metabolites-1353572-supplementary.pdf]
